# Remote realities: In-depth conversations on antimicrobial stewardship in remote Indigenous communities using a One Health approach

**DOI:** 10.1016/j.onehlt.2026.101494

**Published:** 2026-06-23

**Authors:** Anna E. Sri, Kirsten E. Bailey, James R. Gilkerson, Laura Y. Hardefeldt

**Affiliations:** a Asia-Pacific Centre for Animal Health, Melbourne Veterinary School, Faculty of Science, University of Melbourne, Parkville, VIC 3010, Australia; b National Centre for Antimicrobial Stewardship, Department of Infectious Diseases Melbourne Medical School and Melbourne Veterinary School, University of Melbourne, VIC 3010, Australia

**Keywords:** One health, Antimicrobial stewardship, Antimicrobial resistance, Qualitative research, Health systems, Healthcare, Antimicrobial use, Transdisciplinarity, Equality, Equity, Animal health, Environmental health, Human health, First nations, Aboriginal people, Indigenous people, Indigenous health, Australia, Remote communities, Social determinants of health

## Abstract

A One Health approach encourages transdisciplinarity and equity when working to optimise the health of people, animals and the environment. It is helpful to use a One Health approach to explore issues such as antimicrobial resistance (AMR), where a traditionally medicalised focus may otherwise prevent investigation or understanding of lesser known but still relevant influences. In-depth interviews with fourteen participants (nurses, doctors, Aboriginal health workers, veterinarians and antimicrobial stewardship (AMS) pharmacists) were conducted to explore under-investigated topics of AMR and AMS in remote communities as they relate to medical and veterinary practice. We aimed to explore the relationships between factors influencing antimicrobial use and health systems, such as the social determinants of health. Staff shortages and high workload were identified as factors that influenced AMS and the capacity of prescribers to make more appropriate antimicrobial choices. Generally low antimicrobial use in remote community veterinary practice was identified, however veterinary surgical prophylaxis is an area where the quality of prescribing could be improved. Other enablers for AMS include the positive influence of local Aboriginal health practitioners and their equivalents in animal health, openness by participants to improve their practices, and evidence amongst participants of understanding or acknowledgement of upstream drivers of AMR and interest in working collaboratively across disciplines.

## 1 Introduction

The threat of antimicrobial resistance (AMR) has been well-described [1], [2]. The impact of AMR also intersects with other inequalities and social determinants of health such that AMR can be seen as a socio-political problem [3], [4]. In remote Indigenous communities in Australia, the likelihood of people contracting resistant infections such as methicillin-resistant *Staphylococcus aureus* (MRSA) or extended spectrum beta-lactamase *E. coli* (ESBL *E. coli*) can be significantly higher than in urban centres around the country [5], [6]. There is a lack of data from remote Indigenous communities on health practitioners' attitudes and practices towards AMR and antimicrobial use and the specific challenges they face when prescribing in remote settings.

This research aims to increase understanding of how antimicrobials are used in remote Indigenous communities and the broader issues influencing decisions about use in people and animals. To understand more about this complex, cross-sectoral issue, we used a One Health approach. One Health incorporates the philosophy of epistemological pluralism, to create space for principles such as social justice and equity to be highlighted, rather than a reductionist approach focusing only on technical aspects of AMR [7], [8], [9], [10], [11], [12]. Principles of One Health, in particular transdisciplinarity, equity and engagement of communities and marginalised voices are also consistent with some of the aims of Indigenist Health Humanities research and strength-based approaches [7], [13]. Indigenist Health Humanities is a field of research that aims to rectify current gaps in approaches towards researching and addressing health, particularly in relation to inequalities in health and outcomes for Indigenous and non-Indigenous people. It highlights ways of knowing that are relational rather than hierarchical. It encourages consideration of the limitations of some knowledge systems and the need to assess the knowledge created from different perspectives [7]. Indigenist Health Humanities and a One Health approach encourage inclusion of a broad range of stakeholders, as is the aim of the methodology used in this research. This approach increases the likelihood that diverse perspectives and ideas will be considered.

## 2 Methods

In-depth semi-structured interviews with animal, human and environmental health practitioners were performed over an 11 month period from July 2023 to June 2024. Practitioners were included if they were currently working, or had worked in the past 10 years, in a remote Indigenous community. Purposive selection was used to identify participants across a wide geographic area and to ensure input from participants across different professional roles, for example, nurses, doctors, Aboriginal health workers, veterinarians and AMS pharmacists. Recruitment of participants occurred by contacting health services or other relevant organisations, and individuals known to our researchers via phone and email. This sampling methodology was used to enhance the benefits of qualitative research by allowing selection of cases that would provide an increased depth of understanding, particularly for sectors and from individuals who may not have had their views heard previously [14]. Semi-structured interviews were used to obtain rich and complex data. An interview guide (supplementary materials) was created and adjusted for relevance according to the role of the participant being interviewed. Questions included, but were not limited to, those about which antimicrobials were regularly prescribed, access and overuse of antimicrobials, links between AMR in people and animals, impact of AMR on the environment or another health sector, adherence to prescriptions, and the influence of relationships on prescribing decisions. Antimicrobial use described in the interviews was recorded and grouped according to the rating system developed by the Australian Strategic and Technical Advisory Group on AMR (ASTAG). The ASTAG rating system classifies antimicrobials as low, medium, or high importance based on the severity of impact if resistance developed to that antimicrobial.

Interviews were conducted online and were recorded via Zoom video conferencing software. Interviews were transcribed by the primary researcher (first author AES) as part of the data generation and analysis process. Transcripts were coded inductively with a reflexive approach and analysed using thematic analysis and aspects of grounded theory. Information such as place and organisation names that could identify participants were removed from transcripts to preserve anonymity and confidentiality. This allowed participants to speak more freely. As AES had worked in remote communities as a veterinarian, this also helped participants to feel more comfortable knowing that the researcher understood some of the context to their experiences. Some participants were also previously known to AES. AES practiced reflexivity to position themselves in relation to the data generated and described how their personal experiences influenced interpretation of participant responses [15], [16].

### 2.1 Reflection

AES visited and had an ongoing connection to some remote Indigenous communities as a child due to her mother working as a teacher in North-East Arnhem Land for a period of over thirty years. As a teenager and adult, AES worked in education and then later veterinary roles in remote Indigenous communities in North-East Arnhem Land and the Roper Gulf region. Critically, some of this work occurred before and then during the initial years of the Northern Territory intervention during which AES witnessed increased community policing and less support for Aboriginal-controlled community governance. This, along with the consistent activism of strong community leaders, influenced her view of the importance of sovereignty, self-determination and language in maintaining resilience despite the ongoing harms of colonisation. Knowledge of One Health and AMR motivated AES to conduct this research in an attempt to understand and highlight aspects integral to AMS that may be missed by more quantitative and less holistic methods. Her previous research had emphasised the importance of access to diagnostic tests to improve AMS. However, from her experience in remote communities AES knew diagnostic tests were generally not readily available due to costs, lack of awareness and other challenges related to sample collection, storage and transport. AES wanted to focus on principles of equity and transdisciplinarity that are generally not emphasised in veterinary research yet still impact issues such as lack of access to diagnostic tests.

## 3 Results

Fourteen interviews were completed. Interviews ranged in duration from 17 to 63 min with a mean of thirty-five minutes. Participants included five veterinarians, two nurses, two Aboriginal health workers, three AMS pharmacists and two doctors. Most participants worked, or had previously worked, in remote communities in the Northern Territory or North Queensland. Most of the AMS pharmacists included worked externally to remote communities, but their work supported health services operating in those areas, so their perspectives were included. Data saturation occurred once numerous participants in each role had been interviewed and consistent themes were revealed within professions but also across the range of participants without the introduction of new themes.

The key themes identified from veterinarian interviews included difficulty maintaining sterile instruments and surgical conditions generally as well as lack of access to diagnostic tests due to remoteness and costs. Both animal and human health participants used prophylactic or precautionary prescribing more than they would in a non-remote context due to perceived serious consequences and lack of access to subsequent care if a complication developed or a condition worsened. Workforce issues were raised by nearly all participants including challenges such as high staff turnover, staff shortages, and lack of capacity for future planning and training. Another common theme was the importance of education. Participants mentioned education of human and animal health staff about AMR, education of patients and the wider community including school children about preventative health strategies for themselves and their animals, and also for owners about how to manage common minor conditions in their pets. The role of personal relationships also emerged from the data and interacted with other themes such as education and precautionary prescribing.

Antimicrobials used by participants in human and animal health are listed in Table 1. There was significant overlap between the antimicrobials used for both humans and animals, however no high importance-rated antimicrobials were reported to be frequently used in animal health. In both human and animal health, common reasons for antimicrobial use were skin and soft tissue infections, including wounds. Other common uses mentioned in human health were to treat respiratory infections, urinary tract infections, sexually transmitted infections, otitis externa and media, and sepsis. Veterinary participants described that most antimicrobials used in animal health were for surgical prophylaxis. Other common uses mentioned in veterinary health were to treat generally unwell animals, parvovirus, and ehrlichiosis cases.

**Table 1:** Antimicrobials mentioned by practitioners that they usually used or had in stock.

Antimicrobial Class	Antimicrobials Used in Human Health	Antimicrobials Used in Animal Health	Importance rating
Narrow spectrum penicillins	Benzylpenicillin (pen g) Phenoxymethylpenicillin (pen v)	Benacillin (procaine penicillin, benzathine penicillin, procaine hydrochloride) Duplocillin (procaine penicillin and benzathine penicillin)	Low
Moderate-spectrum penicillins	Amoxicillin	Amoxicillin	Low
Antistaphylococcal penicillins	Flucloxacillin		Medium
ß-lactamase inhibitor combinations	Amoxicillin/Clavulanic Acid	Amoxicillin/Clavulanic Acid	Medium
ß-lactamase inhibitor combinations	Sulfamethoxazole/ trimethoprim (co-trimoxazole) (Bactrim)	Trimethoprim sulfadoxine	Medium
Nitroimidazoles	Metronidazole	Metronidazole	Medium
Macrolides	Azithromycin		Low
1st Generation Cephalosporins	Cefazolin (sometimes with probenecid to extend duration)	Cephalexin tablets	Medium
3rd Generation Cephalosporins	Ceftriaxone	Cefovecin (rarely used)	High
Tetracyclines	Doxycycline	Doxycycline Oxytetracycline (alamycin)	Low
Glycopeptides	Vancomycin (especially if suspect MRSA and for sepsis pack)		High
Carbapenems	Meropenem (for sepsis)		High

The primary driver for antimicrobial use for surgical prophylaxis in veterinary medicine was inadequate sterilisation of surgical instruments. Veterinarians were using sterilisation methods such as boiling, baking, or using disinfectants such as chlorhexidine rather than the standard autoclave technology used in most veterinary practices in Australia. Veterinarians were using other tools to minimise the risk of surgical contamination, such as using drapes, but the lack of autoclave capacity was perceived to increase the risk of surgical site infection.

*“If I could sterilise every kit before I use it, then my surgery technique would be as good if I was at home because we do… everything gets scrubbed…everything gets a drape and things get sterile swabs. It's literally just the kits that we don't have ability to cook everything.”*

*(Veterinarian, participant 9)*

*“It'd be nice to have the funding to put an autoclave in each community and then we wouldn't have to worry.”*

*(Veterinarian, participant 5)*

However, one participant had found that prophylactic antimicrobials were generally not required for their surgeries even without autoclave sterilisation.

*“I don't give any antibiotics for surgery at all... the methods that I'm using for my surgeries, I feel quite confident with infection rates and things like that… it's got to be pretty bad for me to send it with antibiotics post-op.”*

*(Veterinarian, participant 1)*

Treatment of superficial wounds with topical treatment and wound flushing was also described by veterinarians, with all positive experiences including for deep wounds. This therapy is consistent with the recommendations of current Australian veterinary antimicrobial prescribing guidelines [17].

*“We've got our first aid cabinets in each community which have got wound flush packs with chlorhexidine [an antiseptic and disinfectant] and antibiotics and metacam [pain relief medication] and stuff. Then we will just hand out a wound flush pack and not hand out any antibiotics at all. So yeah, if it's a fresh wound, then we try to just treat it with chlorhex rather than adding in the antibiotic.”*

*(Veterinarian, participant 5)*

Another issue faced by veterinarians was lack of access to diagnostic tests, particularly culture and susceptibility testing, due to remoteness and costs.

*I've never done it to be honest because I guess then there's the thing of, ideal world you'd want to do culture and sensitivity on every single thing, for the cost, who's going to pay for that? Cause in community it's [government] and council funded money. I'm not paying for a $300 culture and sensitivity for my own interest and the council's not going to pay for that for anyone.*

*(Veterinarian, participant 1)*

*“I think it would have been feasible through the local health centre, the main challenge tends to be that there's such high staff turnover that if you do establish a relationship with people working there that would allow you to throw in a sample with anything that they're sending off, maybe those people won't be there in a months' time and then you're back to square one of trying to talk them around to allowing you to do that stuff during which time an animal is harboring a potentially nasty antimicrobially resistant infection.”*

*(Veterinarian, participant 2)*

In human medicine, samples were regularly taken for culture and susceptibility and in some cases were used to change the course of antimicrobials given to that patient if resistance was detected, but these results could also be used to inform development of antibiograms and understand resistance patterns more generally. None of the participants reported that they used culture and susceptibility testing to change the antimicrobial prescribed to a lower importance rated antimicrobial if the initial empirical treatment was of higher importance.

Prophylactic prescription of antimicrobials was practiced in both human and animal health, perhaps to a greater extent due to the remote locations and perceived consequences of not treating. This also intersected with workforce issues– not being able to be present for follow up care, and clinical confidence to withhold antimicrobials.

*“Definitely the first few years I was in community, I mean even just the first few years as a vet, I think you just give it some amoxyclav [amoxicillin clavulanic acid] because what if that gets infected and that owner comes in and complains to my boss that ‘I came in, and that young vet didn't give me antibiotics.’ Whereas now I'm like, ‘It'll be fine, go away’.”*

*(Veterinarian, participant 1)*

*“I have that sort of moral dilemma of whether or not I'm gonna give it to the animal, it's more based on whether the animal would get sick and I'm not going to be here to be able to see it because I leave this afternoon and there's no vet for another three months.”*

*(Veterinarian, participant 4)*

For both human and animal health practitioners, considering whether a course of antimicrobials could be completed by the patient or administered to the client's animal influenced whether these were prescribed. There was consideration of numerous factors including social determinants of health when making these prescribing decisions.

*“I don't know if it differs to non-remote communities, but certainly compliance and adherence to medication overall is often an issue. I guess culturally, sometimes you know, it's about health literacy, making sure that people understand why they need to take this medication and why they need to take it every day and sometimes multiple times a day. So conferring that understanding, which sometimes may or may not occur with some of our clients because their literacy is low. English literacy is low already, so certainly that presents an issue with understanding, first off, so I think that kind of plays in a bit with some of that also, I guess culturally, sometimes there are other issues in someone's life, and I feel like the social determinants of health kind of play into that as well. Like, if someone's homeless or doesn't have, you know, security, where are they gonna put their medications, if their medications need refrigeration, they don't have a fridge, so you know, medication security is an issue as well. So I think there's a few different factors at play that may impact someone's adherence that sometimes may not be looked at.”*

*(AMS pharmacist, participant 13)*

*Sometimes the dog's not there, or sometimes the owner is asleep for the morning so it doesn't get the morning dose. And there are obviously cultural things that take precedence understandably so one owner might be there one day and the next day they've gone over to another community for ceremony and that sort of thing. There's a lot less predictability in where they might be, where the dog might be, so that is more what stops me from prescribing courses of antibiotics and I guess I'm conscious of tablets not being used and being left there and being used in a different capacity, being picked up by little kids and eaten or if it's just used in a way that they shouldn't be because they're a tablet so I suppose from a compliance point of view not getting into the dog is one thing but I guess misuse, not necessarily in a naughty way, just accidental and obviously having antibiotics lying around and going into the bin and going out to the landfill and that sort of thing isn't particularly ideal either. If I think that an owner isn't going to be compliant I don't think that's necessarily because of the communication error that could be resolved by talking to somebody else that is local to translate that, yeah. I think if that was the case I would be relying on those resources that we do have to communicate that if I thought that that was the main problem.”*

(Veterinarian, participant 4)

Views around overuse of antimicrobials were mixed. Some participants thought the risk of consequences or lack of access to other safeguards justified more frequent use of antimicrobials than in urban practice.

*“Any sore throat, even if it's COVID, gets LA bicillin injection [long-acting penicillin] and potentially also tablets like phenoxymethylpenicillin to treat for possible strep”*

*(Doctor, participant 6)*

*“In our population in remote communities, I think that the risk of rheumatic fever and post strep GN [glomerulonephritis] is incredibly high for those kids with absolutely disastrous potential outcomes. And so I don't think that there's overuse, but I do recognise that outside of that specific environment, then a lot of the antibiotic indications are not necessarily global indications.”*

*(Doctor, participant 6)*

Some of the consequences that concerned prescribers were not just medical but had flow-on impacts related to trust and preserving good relationships with community members.

*“Recognising that relationships with community members are something that needed to be protected and that if surgical cases didn't go well for something as simple as a post-operative infection, that that could really set the care of all animals in that community back due to mistrust.”*

*(Veterinarian, participant 2)*

*The more well known you are in a community, the more trust typically you will have… But then it can go both ways, right? Like if you upset somebody or if you are like looking after a patient who dies in hospital…there potentially will be a lot of mistrust, and that extends far and wide. It's not just this four-person family unit will have mistrust. It's suddenly one hundred people. So yeah, it goes both ways.*

*(Doctor, participant 6)*

Another participant thought increasing levels of resistance in human health indicated that current prescribing practices were not appropriate.

*“We see high resistance in remote settings and so a very, very black and white example is MRSA. In [location] the overall rate is 53% of all Staph aureus isolates and that's crazy. Much, much higher than anywhere else in Australia. I believe that's predominantly caused by overuse of antibiotics. Our ESBL rate has gone up from about 7% back in 2013 to 23% back in 2022. I was dumbfounded by this ‘cause [they're] usually low but within a state of 10 years’ time, it's just rocketed, just skyrocketed. So we must have been doing something wrong.”*

*(AMS Pharmacist, participant 11)*

*“I think a huge problem is because people are really far away, right, so when they're a bit sick, when they're sick, when they've got respiratory illnesses, a lot of the time, the easiest way is just to give them a shot of ceftriaxone and then send them home. And then keep doing it until if it's not improving then they'll send them to the hospital. If they're improving, then they'll just finish the course. I can understand the mentality, but I cannot accept it because this is really, really poor use of medication and this is something that we're trying to address as well.”*

*(AMS Pharmacist, participant 11)*

Although views were highly variable, human health practitioners with the exception of Aboriginal Health Workers, did not consider the impact of AMR or zoonotic and zooanthroponotic disease transmission as much as veterinarians did, or when they did, only considered animal to human transmission.

*“We keep getting told no, there's no link between the dogs and people having scabies. You can't help feel that something's not right. So whether it's… yeah I don't know. Again, I would seek clarification if there's a cross going over, cross-over from dogs to people. The other one we had recently, in the last few months, was fungal infection, kerion, which is a scalp one, tinea capitis, and apparently dogs are associated with passing that on. But we've only had one confirmed case – one or two.”*

*(Nurse, participant 3)*

*“When I was working remote, I think you don't really think about it [AMR] so much, more trying to think about the individual patient.”*

*(Doctor, participant 14)*

*“I haven't had that sort of discussion with people before [about the impact of AMR on animals and the environment]. And the fact that I've never, not really, had that discussion with people before certainly illustrates that it's not high on my uh, it's not in the forefront of my thoughts. And yeah, it hasn't come up much in conversation, so maybe not in others as well.”*

*(AMS Pharmacist, participant 10)*

However, many participants mentioned upstream factors that would impact on the development of AMR or influence how they prescribed such as housing, education, and environmental factors such as heat and humidity.

*“It's like overcrowding sometimes. Problem you know, people staying in one house – too many people, and that's why people get like scabies and skin infections. And I go there and give education about how to look after your skin or come to the clinic like for checkup, and that's part of my work.”*

*(Aboriginal Health Worker, participant 12)*

*“I think the housing and the fridges is …that's actually quite a big one…pretty much everything [medication], after you reconstitute it, is generally kept in the fridge…and then sometimes…you're having to reconstitute multiple times and depending on the course and the duration and things. It's not necessarily straightforward for some families who are not all that health literate.*

*(Doctor, participant 14)*

Aboriginal Health Workers were very aware of the impact of animals and the environment on human health and emphasised the need to care for animals.

*“I always think about it. I have to tell to not do this, or so we have to, like, clean the houses, all that stuff, leave the dog outside, not inside, not in your kitchen area or in the bed. This kind of story we have to educate them over and over.*

*(Aboriginal Health Worker, participant 8)*

*People love animals. But they need to look after the animals like doing the washing or seeing the vet like to give like a medicine or worm tablet for the dog or you know skin… give ivermectin. Also I talk to the people about how to look after pets, you know animals, pets, dogs but sometimes people they don't like dogs because they scratch a lot on the mattress or blanket and the children you know come and sleep on the mattress and sometimes they feel itchy.”*

*(Aboriginal Health Worker, participant 12)*

Staff shortages and high workload were regularly mentioned by participants. Workforce issues influenced how some participants prescribed antimicrobials. Concerns included the time period before healthcare services would be available again and the high expectations placed on staff.

*“We might only go out there once a week or once a fortnight, so if I had someone presenting with skin sores I would rather treat them then, than wait two weeks to see if they resolve on their own.”*

*(Nurse, participant 3)*

*“A lot of the communities are working a lot on agency nurses. There's not many people staying permanent in community, the nursing and the doctors… the same as [remote community location] they haven't got a permanent doctor. It's just locums. Sometimes you work without a doctor…and that's quite a big…becomes a bit weary as well.”*

*(Nurse, participant 7)*

Planning and future training were also impacted. Barriers for new staff joining included training and onboarding requirements and a lack of time made available for this.

*“So we have been giving…delivering talks, but I think again it's not just one problem. We're trying to deal with multiple issues, for starters staffing issue, like the workforce is so transient…you know who you get today might not be the person you get tomorrow. You also have the doctors versus nurses versus Aboriginal health workers and also it's so sparsely, you know, it's so spread out so when you run education sessions, you're never ever going to capture everyone. The talks that you give to doctors have to be pitched differently to the talks that you give to nurses. It's just difficult.”*

*(AMS pharmacist, participant 11)*

*“You kind of go down the path of training them and then they just don't turn up to work. I get approached by people on a weekly basis all the time saying that they wanna come and work for us but when it comes to the nitty gritty of, OK, you've gotta go and fill in forms and you gotta do the paperwork and you gotta do this and that…they're just not there… It's all just kind of a bit frustrating and unreliable.”*

*(Veterinarian, participant 5)*

*“I think yes, it's hard for them. If they come to doing the course in Darwin, like training and all these courses.”*

*(Aboriginal Health Worker, participant 12)*

Nearly all participants mentioned education programs as a way of addressing AMR and infectious diseases.

*“Why don't we do more in that space [bush medicine, home remedies], if your dog is sick, these are things you can do. If that doesn't work, the vet can help you, and like education and getting into the schools and doing that kind of thing.”*

*(Veterinarian, participant 1)*

*Mostly… I spoke about, not about the tablets or medication, it's all about like education, how to look after yourself, how to look after the house, outside the house or inside the house, make sure it's clean… Sometimes I hand over the posters about healthy homes and stickers – clean the house, make sure that people come and see posters about healthy homes. And also I give education about scabies or crusted scabies, Strep A, germ bacteria, hygiene. Yeah, all those kinds of stuff here.”*

*(Aboriginal Health Worker, participant 12)*

In some cases, there were concerns about carrying out education programs because an appropriate action or solution was not currently easily accessible to community members (in this case vaccination to prevent canine parvovirus).

*“We're currently in decisions and we've just had a parvo outbreak in [remote community location] two weeks ago, which is currently in [remote community location] as well and has made it into [town] with private animals. We can't run an education programme on it without being willing to back that up with vaccinating. So then it's the argument on are we going to offer vaccination. So we'll see. Time will tell.”*

*(Veterinarian, participant 5)*

There were varying levels of importance placed on working with local staff. In some cases, the focus was on completing urgent tasks rather than local employment or training.

*“I found I spent more time running around trying to find the person that was meant to be helping me than actually doing any work or doing the stuff they were meant to be helping me with. So I kind of gave up a little bit.”*

*(Veterinarian, participant 5)*

Despite this, some participants consistently expressed how important it was to have local people working in healthcare.

*“Yeah, have a lot of respect for them, love working with them. It just brings a better feel to the clinic there. So much more knowledge. They just know who's where, so helpful. It's a lot harder without them.”*

*(Nurse, participant 7)*

*“It's great to work with them. They're an amazing resource and you know a lot more trusted in the communities than often we were. As we just fly in and fly out for one day a week. So yeah, they were really amazing to work with.*

*(Doctor, participant 14)*

*If you didn't have that connection with the local workers, it would be more difficult. It would be harder. It would literally go back to just picking up dogs, desexing them and letting them go again.*

*(Veterinarian, participant 9)*

Aboriginal health practitioners stressed the importance of their role. The influence of role models in encouraging people to become health practitioners was also mentioned.

*“If people come, they come to us to see nurse or doctor and as an Aboriginal Health Practitioner, we have to be there as well. So we can explain in our language. Understand what the antibiotic is for – don't have miscommunication with nurse or doctor. So it's important to have an AHP [Aboriginal health practitioner] there, to be present.”*

*(Aboriginal Health Worker, participant 8)*

*Because my mother she was a health worker. Like she was my role-model.”*

*(Aboriginal Health Worker, participant 8)*

Participants stated that a transitory workforce made it more difficult to build trust and relationships with community members.

*“I think those relationships are certainly really important and it's tricky when we have revolving…and the same with our health centre nursing staff as well, there's a lot of locum workers.”*

*(AMS pharmacist, participant 13)*

Participants who had spent a longer time in communities reported better relationships and interactions.

*“We've built that over years and we're at a point where people understand about antibiotics and understand about looking after their dogs and what the vet does and that kind of thing.”*

*(Veterinarian, participant 1)*

*I think that's part of the reason why it's important to…if it's suitable for people to stay for a long time because the more trust you develop, the better. It just takes time.*

*(Doctor, participant 6)*

Some practitioners had a desire for self-improvement and also improved standard of care. They expressed frustration at the lack of buy-in from other sectors and that some health practitioners were not trying to improve their own practice, instead blaming external conditions.

*“It's not a problem that can be resolved with just one plan, like, you know with one strategy. You need to have everyone on board. I actually thought that we are in a pretty bad situation… bad enough to instigate, you know, more buy-in from people, but apparently not. So I don't know.”*

*(AMS pharmacist, participant 11)*

*“It's like ‘oh well, you know, it's community, it's dirty, we're going to give antibiotics’. And I'm like, ‘but you're dirty!’ [laughs] You know better, you have access to education, you could do better”*

*(Veterinarian, participant 1)*

## 4 Discussion

This study highlights the challenges faced by veterinarians without access to infrastructure and equipment such as clean surgery rooms and autoclaves, and other drivers of antimicrobial use and infectious disease such as housing shortages and overcrowding. These findings were expected from the personal experiences of the primary researcher (AES) working in remote Indigenous communities and from the results of other studies [18], [19]. Previously identified barriers to animal health in Indigenous communities, including remoteness, accessibility and funding limitations [20], were also identified by participants in this study in relation to the use of antimicrobials. However, the use of this qualitative One Health approach highlighted themes not often discussed in veterinary science yet which impact on the development of AMR and implementation of AMS strategies. Some of these include the impact of workforce issues, the influence of relationships and trust with community members, and, particularly in veterinary practice, reliance on informal relationships and strategies to enable treatment of animals or diagnostic testing. Using qualitative semi-structured interviews to gain deeper understandings of AMR and AMS has proven beneficial in previous research with pet owners [21], [22]. We found in this case, it provided a safe setting to discuss the issues in a relaxed environment without time pressure.

The issue of workforce has been discussed in the context of AMS [22] but the focus has been on interactions with colleagues and clinical experience. In this case staff shortages, as well as high rates of turnover and overwork generally, may be a significant and potentially overlooked factor in the fight against AMR. Our participants highlighted that providing education and training about AMS to other staff was difficult given staff turnover. Due to having limited time in the communities, there was more motivation to prescribe antimicrobials to prevent infections when no health practitioners would be available to provide follow up care. Given the cost of AMR into the future, funding workforce sustainability could be an investment in AMS. Enablers for AMS include the positive influence of local Aboriginal health workers in human health and their equivalents in animal health, a strong interest by participants to improve their practices or receive more knowledge about aspects of AMR, and evidence amongst all participants of at least some understanding or acknowledgement of upstream drivers of AMR. Transdisciplinarity and collaboration between sectors is critical when dealing with issues such as housing and environmental health [6]. Our results suggest this needs to go further to include animal health and a focus on increased engagement and employment of local people, along with sufficient resourcing.

Direct questions about racism or discrimination were not asked, although there was recognition by one participant that negative experiences by locals and media reports about a particular hospital may have influenced how community members sought healthcare. There was little self-reflection by non-Indigenous participants about how race and discrimination may impact access to antimicrobials or interactions between health practitioners and local people, particularly as adherence to medications and late presentation were mentioned frequently [23]. There have also been recent media and organisational reports about the impact of racism on healthcare in Australia and internationally including as part of “Black Lives Matter” campaigns [24], [25], [26]. Participants may not have felt comfortable to raise issues of racism themselves or did not feel it was relevant in AMS research. It cannot be determined whether issues of racism were unsaid because it was not occurring, participants weren't comfortable to raise them, or because participants were unfamiliar with systemic racism and microaggressions. A lack of awareness, particularly of microaggressions, may have contributed to participants not even recognising their potential impact. Epidemiology and health research has been described as, “A discipline so neutral it cannot, or rather will not, account for state-sanctioned violence, oppression, and dispossession in ways that could describe, explain, predict, and control inequity” [7]. Participants did, on occasion, refer to unspecified cultural factors in impacting health-seeking behaviour but did not explore or define this further. Participants may be using this term to describe barriers caused by racism or other discrimination without explicitly naming them, however this still allows the perpetuation of racist outcomes [27].

There is potential for improvement in animal health through veterinarians sharing knowledge and advocating for improved standards. For example, to local councils who are generally responsible for funding animal health programs and providing infrastructure. Exploring the strategies and outcomes of veterinarians not routinely using antimicrobials for surgical prophylaxis may build confidence amongst other veterinarians to improve their antimicrobial prescribing for surgical cases. A proactive response from the animal health sector also enables more collaboration with the human health and environmental health sector in understanding the barriers and enablers in each section and working together collaboratively where possible. Many community health programs already incorporate aspects of One Health such as prevention of zoonotic diseases and improving access to WASH (water, sanitation and hygiene) facilities. Some participants described being involved in such programs incorporating input from other health sectors.

Numerous researchers have mentioned the importance of involving and working with local staff on animal health programs [18], [28]. Our research also identified the difficulties that health practitioners, especially veterinarians, had in increasing the skill level of the local workforce in managing animal health in the absence of a veterinarian in the community. The time taken to strengthen workforce capacity while focusing on tasks perceived as more urgent such as surgery was mentioned by numerous participants and has been identified previously [28]. In the context of AMS education, this may be further exacerbated by the complex nature of AMR and its impacts, which are not often well-understood by the general public [21]. An evaluation of short-term benefits in conducting animal health programs versus the long-term benefits of increasing local capacity would be beneficial.

While many participants mentioned education as a solution to health challenges, we must remember that we don't necessarily ‘educate’ our way out of health issues in mainstream communities. Behaviour change often requires more than education alone, demonstrated in AMS interventions such as colour-coding antimicrobials and moving high importance antimicrobials to less accessible parts of the pharmacy [29] as well as in programs for other chronic health behaviour change interventions [30]. Lessons from the COVID-19 pandemic show that social determinants of health had a significant impact on public health that could not be mitigated by provision of education alone [31], [32]. If people do not have reliable access to fundamental needs such as clean water, food and housing, education about handwashing or avoiding over-crowding may not be helpful [33]. Participant responses suggest that there is a tendency for health practitioners to focus on the ‘solution’ of education, rather than addressing upstream factors, potentially due to the perceived enormity and persistence of current inequalities and associated challenges.

There has been limited qualitative One Health-focused research in the field of AMS in Australia, particularly as it relates to remote communities. Active inclusion of Indigenous viewpoints is recommended by others [20] and our study benefited from their perspectives, with three out of fourteen participants identifying as Indigenous. There is potential to increase the involvement of more Indigenous participants, however care must be taken to ensure a One Health approach does not solely address pre-identified health problems without centring the needs of the communities, whose priorities may differ from those of non-Indigenous academics or health practitioners [7]. If research does occur, participatory and co-designed approaches to ensure activities are conducted in alignment with community priorities and needs should be the minimum standard [34]. Focusing on existing strengths, successes, and enabling factors may help avoid burdening Indigenous health practitioners, community leaders and traditional owners with additional responsibilities related to research [8]. For example, providing additional support for Aboriginal Health Workers to engage in research or targeted training in areas of interest to them [35], [36], and providing financial and mentorship support to young people wanting to train as Aboriginal Health workers or practitioners.

## 5 Conclusion

This research highlights the current strengths in remote communities in relation to AMR prevention, as well as the drivers of AMR and their interconnection with upstream factors. This research can guide discussions on how remote communities would like to engage with AMR and existing AMS approaches, such as use of prescribing guidelines and educational materials. AMS efforts must move beyond focusing on individual behaviours of community members or health professionals to addressing upstream social, economic, cultural, legal, linguistic, and political conditions that underlie health inequalities and drive and maintain AMR.

## CRediT authorship contribution statement

**Anna E. Sri:** Writing – review & editing, Writing – original draft, Visualization, Project administration, Methodology, Investigation, Formal analysis, Data curation. **Kirsten E. Bailey:** Writing – review & editing, Supervision. **James R. Gilkerson:** Writing – review & editing, Supervision. **Laura Y. Hardefeldt:** Writing – review & editing, Supervision, Methodology.

## Informed consent statement

Informed consent was obtained from all participants in the study.

## Ethics

The study was conducted according to the guidelines of the Declaration of Helsinki, and approved by the Human Research Ethics Committee of the Northern Territory (NT HREC reference number 2023-4519) and the Human Research Ethics Committee of The University of Melbourne (Ethics ID number 2023-25366-36024-3).

## Funding

The research was funded by the Australian Research Council through the Discovery Early Career Research Award Program (DE200100030). L.H. was funded by the Australian Research Council through the Discovery Early Career Research Award Program (DE200100030). A.S. was a recipient of an Australian Postgraduate Award scholarship.

## Declaration of competing interest

The researchers declare that they have no known competing financial interests or personal relationships that could have appeared to influence the work reported in this paper.

## Data Availability

Data will be made available on request.

## Appendix A Supplementary data

Supplementary material: Interview Guide
