# Supplementary material for: Remote realities: In-depth conversations on antimicrobial stewardship in remote Indigenous communities using a One Health approach
Source: One Health. 2026 Jun 23;23:101494. doi: 10.1016/j.onehlt.2026.101494 (PMC13320485; doi:10.1016/j.onehlt.2026.101494)
Supplement: Supplementary file 1 — Supplementary material: Interview Guide [file mmc1.docx]

# Interview Guide for One Health Interviews

**Attitudes to Antimicrobial Stewardship**

**INTERVIEW GUIDE**

**Location:** Zoom

# Introduction

- Welcome, briefly introduce yourself (name, position, organisation)
- Small talk & acclimatization, record participant information
- **Share Patient Information Sheet and Consent** form, talk interviewee through, answer any questions and let her/him sign digitally or in hardcopy if not already done, collect Consent Form
- Inform interviewee (‘Just letting you know that I’m turning on the recorder now’) & **turn on the audio recorder**

# Opening statement

Thank you very much for taking the time to meet with me and doing this interview as part of our project. During this interview today I would like to learn more about your personal experience with antimicrobial resistance and stewardship and hear about your experiences relating to this while working in a remote Indigenous community. Sometimes I might ask you to give an example or describe your thought process. Feel free to take your time and think about it. Remember, I’m interested in hearing your experiences and opinions, so there are no right or wrong answers.

And also, you don’t have to talk about anything you don’t want to. You can stop the interview at any time, take a break, or end the interview altogether – just let me know.

The interview will take about 45 minutes. As mentioned before and on the consent form it is recorded. I would like to emphasize that your responses will be de-identified when transcribed so that your identity and details of your property or locations you might mention will not be known.

For the record – have you received the information sheet, signed the consent form and are you willing to proceed with the interview?

Do you have any questions before we begin?

1. General introductory question enquiring how they came to work in the community and for how long they worked there.

**[For Veterinary Prescribers]**

1v. Can you tell me about which antibiotics you stocked when you were working in the remote communities?

- Did you ever use a third generation cephalosporin like cefovecin "convenia" or fluoroquinolone like enrofloxacin "baytril"?

2v. Why did you carry this range of antibiotics?

- price/donated stock

- ease of administration

- efficacy

- familiarity

- drugs already in stock when started

- other

3v. Do you use antibiotics for surgical prophylaxis? How do you choose which cases need surgical prophylaxis? Route, frequency/time of administration.

**[For All Prescribers]**

1p. Does the way you prescribe antibiotic differ in remote communities compared to other places you’ve worked? How?

2. For what conditions did you mostly use antibiotics and what influences which antibiotic you choose? Do you use antimicrobial prescribing guidelines (ie. Therapeutic guidelines for medical practitioners, AVA guidelines)?

2. Is lack of access to antibiotics an issue that affects human or animal welfare? Do you think there is an overuse of antibiotics in this community or other remote communities that you’ve worked in?

3. Do you think AMR is/was a problem in this community?

Who is it a problem for?

Does it affect how you (or others) prescribe antibiotics?

Can you tell me about the most common diseases you see where AMR is a problem?

4. When you were working in the community did you think much about antibiotic resistance or know much about it?

- the impact on animals

- the impact on people

- the impact on the environment

5. Do you think that there is a link between AMR carriage in animals (ie MRSA in dogs) and AMR in people? Why do you think there is or is not?

Compliance/importance of taking medicines

6. Can you think of any particular tool or resources that could help you when choosing whether to use antibiotics or not or which antibiotic and dose to choose?

7. Can you speak about the impact of relationships in the community and social connections on your prescribing habits?

8. Closing question:

We’ve covered quite a lot – thank you! Is there anything else you think is relevant or important about the use of antimicrobials in remote Indigenous communities?
